# Effects of Hyperbaric Oxygen Therapy in the Treatment of Patients With Central Retinal Artery Occlusion: A Retrospective Study

**DOI:** 10.7759/cureus.66196

**Published:** 2024-08-05

**Authors:** Filipa Maldonado, Alexandre Reis da Silva, Rui A. Ramos, Clara Gaio-Lima, Ana Castro, António Pedro Ferreira, Óscar Camacho, Carla Teixeira

**Affiliations:** 1 Department of Anesthesiology, Unidade Local de Saúde de Matosinhos - Hospital Pedro Hispano, Matosinhos, PRT; 2 Department of Ophthalmology, Unidade Local de Saúde de Matosinhos - Hospital Pedro Hispano, Matosinhos, PRT; 3 Department of Hyperbaric Medicine, Unidade Local de Saúde de Matosinhos - Hospital Pedro Hispano, Matosinhos, PRT

**Keywords:** ophthalmological emergency, visual acuity, treatment outcome, hyperbaric oxygen therapy, central retinal artery occlusion

## Abstract

Background

Central retinal artery occlusion (CRAO) results in sudden, painless vision loss. As an analogous condition to acute ischemic stroke, CRAO is an ophthalmological emergency, but a standardized treatment is lacking. Hyperbaric oxygen therapy (HBOT) has been widely used in spite of the inconsistent results reported.

Purpose

To report the visual acuity (VA) outcomes in all patients submitted to HBOT with non-arteritic CRAO in a tertiary center.

Methods

This retrospective study included all adult patients with CRAO and symptoms lasting for less than 24 hours who were prescribed HBOT in the Hyperbaric Medicine Unit of a Portuguese hospital from March 2009 to February 2023. Patient demographic information, medical history, ophthalmologic evaluation, hospital of referral, time until HBOT, supplementary treatments, number of HBOT sessions, adverse effects, and patient subjective VA gain were collected. All patients were subjected to 90-minute HBOT sessions with 100% oxygen at 2.4 ATA. The primary outcome was VA change (dif-logMAR) before and after treatment. A clinically significant visual improvement was defined as a dif-logMAR≥0.3. Data were analyzed using IBM SPSS Statistics for Windows, Version 29 (Released 2021; IBM Corp., Armonk, New York, United States) (p<0.05 is considered significant).

Results

A total of 114 patients were included in this study; 68% (n=77) were male, with a mean age of 69 years, and were subjected to a median number of seven HBOT sessions. No serious adverse effects from HBOT were reported. The mean time delay from symptoms to treatment was 12 hours, and best-corrected visual acuity (BCVA) at baseline was counting fingers or worse in 84% (n=96) of the patients. A dif-logMAR≥0.3 occurred in 46% (n=52) of the patients, and 58% (n=66) reported subjective VA improvement after the treatment. A significant improvement between BCVA before HBOT (2.12±0.74) and after HBOT (1.67±0.74) was observed. The VA outcome was found to be related to the total number of sessions, age, obesity, supplementary treatments, and cherry-red spot (CRS) at presentation. There were no significant effects of the time delay from symptoms to treatment in the explanation of the VA outcome.

Conclusions

HBOT appears to be safe and has a beneficial effect on VA outcomes in patients with non-arteritic CRAO, particularly depending on the number of sessions. Patient factors such as age, obesity, and the presence of CRSs also appear to influence the VA outcome.

## Introduction

Central retinal artery occlusion (CRAO) is a condition that results in sudden, painless vision loss [1]. The incidence of CRAO is estimated to be 1-2 cases per 100,000 on an annual basis [2,3]. Importantly, morbidity is high, with over 80% of patients having an initial visual acuity (VA) of count-fingers or worse [4], and it has been found that less than 20% of patients obtain functional visual recovery without any treatment [5]. The duration of the occlusion of the central retinal artery is a critical determinant of retinal damage and final visual outcome in CRAO [4]. It has been postulated that retinal infarction may occur after only 12 to 15 minutes of complete CRAO [6]. Studies of CRAO conducted on animal models have revealed that an ischemic insult to the retinal tissue lasting more than 90 minutes results in some degree of damage to the inner retina; if the ischemia persists for more than 240 minutes, the damage is deemed irreversible [6,7]. Thus, CRAO is considered an ophthalmological emergency analogous to acute ischemic stroke.

To date, no consensus has been achieved regarding the most effective method of treating CRAO. Treatments such as ocular massage, hemodilution, intravenous acetazolamide, mannitol, and anterior chamber paracentesis [5,8] failed to demonstrate significant improvements in visual outcomes. Intravenous thrombolysis within 4.5 hours of symptom onset was associated with a higher likelihood of a favorable visual outcome for acute CRAO [5]. However, the evidence to support the general use of thrombolytics in treating acute CRAO remains unclear. The European Committee of Hyperbaric Medicine (ECHM) at the tenth European Consensus Conference on this subject suggested considering hyperbaric oxygen therapy (HBOT) for patients suffering from CRAO, to be applied as soon as possible (type 2 recommendation, level C evidence) [9]. Additionally, HBOT is classified as IIb according to the clinical practice guidelines of the American College of Cardiology/American Heart Association (ACC/AHA) [10].

The rationale for the application of HBOT to treat CRAO is the dual vascular supply of the retina [11]. Under normal conditions, the choroidal circulation provides 60% of the oxygen required for retinal function, and this percentage increases to 100% under hyperbaric conditions [12]. By using HBOT to treat CRAO, there is a potential advantage in that the increased blood flow from the collateral and choroidal circulations can meet the metabolic demands of retinal cells, while the central retinal artery re-cannulates naturally. This can potentially help to preserve the inner retinal layer as long as levels of oxygen in the choroidal circulation are adequately high [13] and irreversible infarction damage has not developed [14]. Nevertheless, the use of HBOT in CRAO remains a field where further research is still needed. A considerable number of case series and clinical research reported that patients with CRAO experienced VA improvement after HBOT [14-18] although there are a few reports that fail to replicate a similar conclusion [19]. It should be noted that most studies were conducted with relatively small sample sizes, exhibited significant variation in the average time from symptom onset to oxygen therapy, and lacked proper exclusion criteria, for cases such as arteritic CRAO or those with patent cilioretinal artery. Despite the recognition that earlier HBOT leads to better outcomes, reliably predicting which non-arteritic CRAO patients will benefit from treatment is also challenging. While recently the presence of cherry-red spots (CRSs) on fundoscopy and baseline BCVA were identified as potential predictors of visual outcome [14], further research is needed to establish reliable predictors of HBOT effectiveness.

The purpose of this work was to report the VA outcomes in all patients submitted to HBOT with non-arteritic CRAO in a tertiary center and to identify factors that may influence these outcomes. This article was previously presented as a meeting abstract at the 47th Annual Scientific Meeting of European Underwater and Baromedical Society on September 14, 2023.

## Materials and methods

Study design

This was a retrospective analysis of patient records with suspected CRAO submitted to HBOT from March 2009 to February 2023 in the Hyperbaric Medicine Unit of Hospital Pedro Hispano in Portugal. All the patients referred to the unit had an evaluation by an ophthalmologist with the diagnosis, confirmed or suspected, of CRAO. This unit is the referral center of approximately half of the Portuguese mainland and CRAO is treated as an emergency.

Data were collected retrospectively from medical records and included the patient’s demographic information, medical history, hospital of referral, fundoscopic findings, time delay from symptoms to HBOT, supplementary treatments, number of HBOT sessions, adverse effects, VA at presentation, VA after HBOT, and patient subjective VA gain.

Referral was considered in two ways: the origin of the referral (Hospital Pedro Hispano or other hospitals) and the distance from Hospital Pedro Hispano (15 km or less, including Hospital Pedro Hispano, or more than 15 km). The limit of 15 km was chosen as the distance threshold, taking into consideration that hospitals located less or equal to 15 km from Hospital Pedro Hispano already have emergency circuits established and so the travel time between hospitals is usually not a significant delay factor. When time delay from symptoms to treatment was not possible to elucidate, such as patients who woke up with visual loss, it was recorded the time of presentation since they last were known to have unaffected vision. Patient subjective VA gain was assessed at the HBOT discharge appointment, by asking each patient if they perceived any enhancement in their VA at the end of the treatment.

The protocol of this retrospective study was approved by the Ethical Committee of Hospital Pedro Hispano.

Hyperbaric oxygen protocol

All patients were submitted to 90-minute sessions with 100% oxygen at 2.4 ATA. The frequency of sessions and the timing of ophthalmological revaluation were dependent on the protocol of the unit. The current protocol was last reviewed in 2018 and consists of two daily sessions in the first 72 hours, after which an ophthalmological revaluation was performed. If best-corrected visual acuity (BCVA) improved, treatment was continued with one daily session until no further improvement was obtained. If BCVA didn’t improve the treatment was suspended. All treatments were performed in the same multi-place chamber.

Patient selection

The inclusion criteria were age older than 18 years and symptoms lasting less than 24 hours. The exclusion criteria were patent cilioretinal artery, no documented BCVA, arteritic CRAO, and branch retinal artery occlusion (BRAO).

Outcomes

The main objective of this study was to verify if the HBOT has a significant effect on VA outcome improvement and identify the other variables that could explain such BCVA outcomes. The primary outcome was a change in the BCVA quantified in the logarithm of the minimum angle of resolution (logMAR) at discharge after HBOT. This change was measured by the difference (dif-logMAR) in BCVA after HBOT (post-logMAR) from BCVA before HBOT (pre-logMAR) which means a post-logMAR value inferior to the pre-logMAR, a negative value on dif-logMAR, indicated improvement. A clinically significant visual improvement was defined as dif-logMAR≥0.3, as an absolute value [20]. VA outcome was expressed by the variables post-logMAR, dif-logMAR, and dif-logMAR≥0.3. In the case of very low vision (<20/400 in Snellen chart), it was assessed by semiquantitative scale, the capability to count-fingers (CF), to see hand movement (HM), light perception (LP) or no light perception (NLP) at a distance of 30 cm. These values were quantified and converted to a numerical form permitting statistical analysis. The decimal values attributed to CF and HM were 0.014 and 0.005, respectively, and the logMAR correspondence adopted was 1.85 and 2.30, as described by Schulze-Bonsel et al. [21]. LP or NLP cannot be retributively converted to decimal values. The values attributed to logMAR for LP and NLP were 2.7 and 3.0, respectively, as reported in the literature [20,21].

Other treatments

Supplementary treatments were considered all the treatments applied prior to HBOT, according to the medical team that evaluated the patient and included non-invasive treatments (e.g., ocular massage, vasodilators, topical and oral hypotensive medication) and invasive treatments (e.g., fibrinolytic therapy, surgical embolectomy).

Statistical analysis

The following statistical analyses were performed: 1) multiple linear regressions (OLS) adjusted on post-logMAR and dif-logMAR and multiple logistic regression adjusted on clinically significant visual improvement (dif-logMAR≥0.3) (binary variable); 2) multivariate analyses of variance (MANOVAs), followed by the Hotelling T2 statistic, on pre-logMAR and post-logMAR to verify the differences between this two variables and the effects of the other variables; 3) Pearson correlation coefficients "r" and Pearson association coefficients "Phi" (binary variables) to verify the relation among all variables. The results were considered statistically significant when p<0.05. The values of “r”>0.185 met such conditions. Data are expressed as mean ± standard error (SE) for pre-logMAR, post-logMAR, and dif-logMAR, as mean ± standard deviation (SD) for other quantitative variables, and as counts and percentages for binary variables as the dif-logMAR≥0.3. All analyses were performed using IBM SPSS Statistics for Windows, Version 29 (Released 2021; IBM Corp., Armonk, New York, United States).

## Results

**Figure 1:**
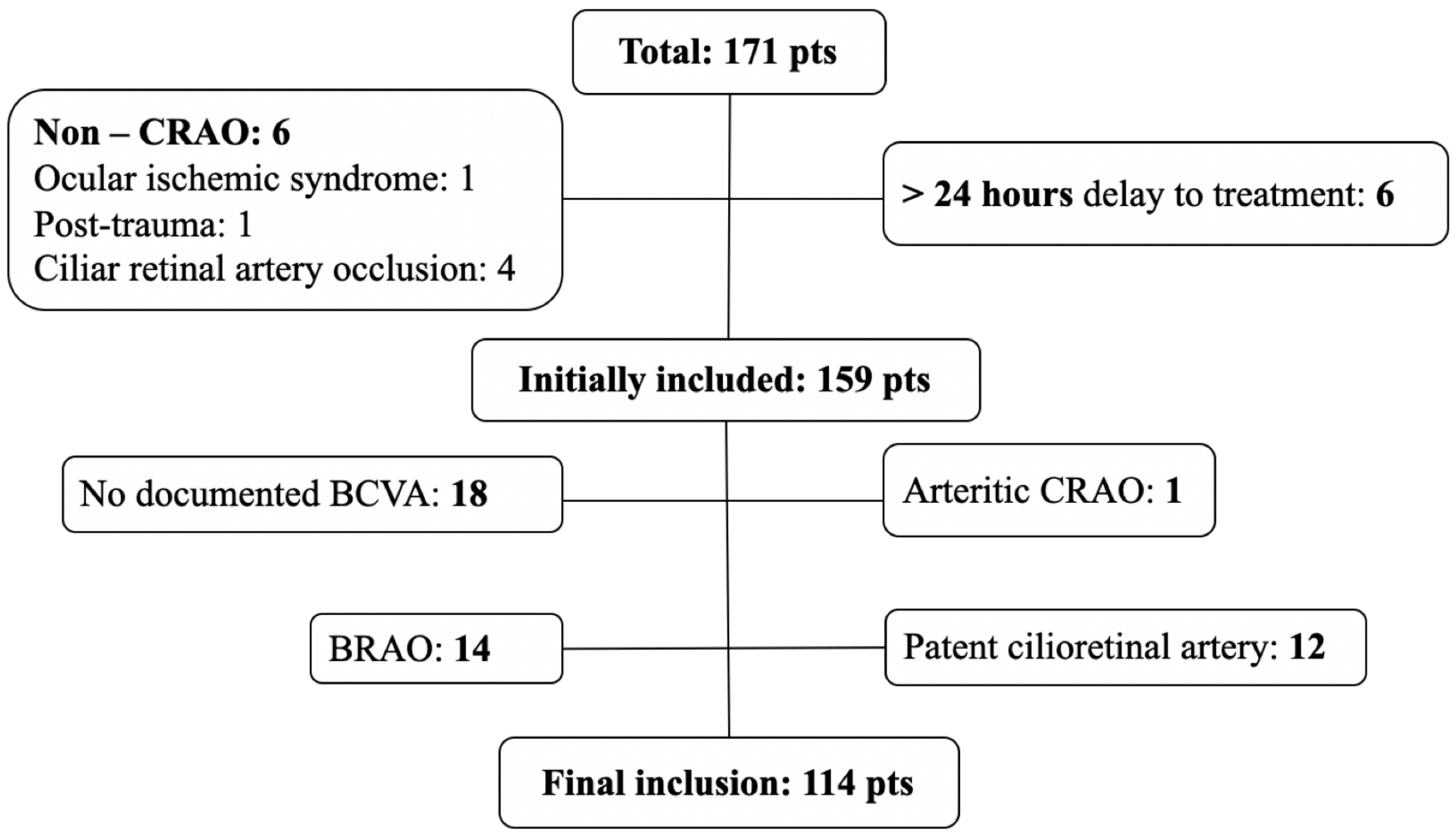
Patients flow Pts: patients; CRAO: central retinal artery occlusion; BCVA: best-corrected visual acuity; BRAO: branch retinal artery occlusion

Of the 171 patients with suspected CRAO treated from March 2009 to February 2023 in the Hyperbaric Medicine Unit of Hospital Pedro Hispano, 114 were included in the final analysis after applying the inclusion and exclusion criteria. Of the total patients, six were misdiagnosed as CRAO and six were treated later than 24 hours from the symptoms. Of the initially included patients 15 had other etiology of retinal artery occlusion (branch or arteritic), 12 had patent cilioretinal artery and 18 lacked documentation of BCVA at baseline or after HBOT (Figure 1).

Patients’ characteristics

Patients’ characteristics are detailed in Table 1. About 68% were male (n=77) and the mean age was 69 years (ranging from 21 to 92 years). The most frequent comorbidities were hypertension and dyslipidemia with a prevalence of 72% (n=82) and 70% (n=80), respectively, followed by cardiovascular disease of 34% (n=39). Prior to the CRAO almost half of the patients were treated with antiplatelets (33%) or anticoagulants (8%) and the majority were doing statins (62%).

Presentation

The mean BCVA at presentation (pre-logMAR) was 2.12±0.74. Of all patients, 84% (n=96) had a VA at baseline of count-fingers or worse (≥1.85 logMAR) and 37% (n=32) had very poor vision acuity at presentation (≥2.70 logMAR) that included patients with only light perception or no light perception. The mean time delay from symptoms to treatment with HBOT was 12 hours (ranging from 2 to 23 hours). About 28% (n=32) of the patients woke up with symptoms. Fundoscopic evaluation revealed the presence of CRSs in 43% (n=49) of the patients at presentation. The majority of CRAO patients were referred from other hospitals (81%). Importantly 54% (n=62) were from a distance equal to or less than 15 km from Hospital Pedro Hispano.

Treatment

The median number of HBOT sessions was 7 with a minimum of 2 and a maximum of 19 sessions. No serious adverse effects from HBOT were reported. Seven patients in total (6%) experienced minor to mild adverse events: three patients experienced mild barotraumas with full spontaneous recovery in a few days; two patients suffered from agitation, irritability, and disorientation without focal neurologic deficits during one of the sessions and fully recovered after depressurization; two patients experienced claustrophobia and were treated with anxiolytics. Supplementary treatments were performed in 46% (n=52) of all patients (Table 1). Non-invasive supplementary treatments found were aspirin, clopidogrel, ocular massage, acetazolamide, ACE inhibitors, brimonidine, statins, timolol, and mannitol. The invasive supplementary treatment found was fibrinolytic therapy. Only five patients, 4% of all patients, were submitted to fibrinolytic therapy.

**Table 1:** Patient’s baseline characteristics, medical history, presentation features, treatment, and visual acuity outcomes BCVA: best-corrected visual acuity; HBOT: hyperbaric oxygen therapy; Nº: number; SD: standard deviation; SE: standard error

Variables	Mean±SD/SE or counts (%)
Age (years)	68.9±13
Sex (male)	77 (68%)
Comorbidities	
Hypertension	82 (72%)
Dyslipidemia	80 (70%)
Cardiovascular disease	39 (34%)
Obesity	35 (31%)
Diabetes mellitus II	30 (26%)
Active smoking	29 (25%)
Ophthalmologic disease	20 (18%)
Neurological disease	16 (14%)
Carotid disease	11 (10%)
Thrombotic disease	4 (4%)
Chronic medications	
Statins	71 (62%)
Antiplatelet drugs	38 (33%)
Anticoagulants	9 (8%)
Presentation	
BCVA before HBOT (pre-logMAR)	2.12±0.74
BCVA before HBOT ≥1.85 logMAR	96 (84%)
BCVA before HBOT ≥2.7 logMAR	32 (37%)
Time from symptoms to treatment (hours)	12.2±6
Waking up with symptoms	32 (28%)
Cherry-red spot	49 (43%)
Referral from other hospitals	92 (81%)
Referral from hospitals located ≤15 km	62 (54%)
Treatment	
Nº of HBOT sessions	7.3±3
Complications of HBOT	7 (6%)
Supplementary treatments	52 (46%)
Visual acuity outcomes	
BCVA after HBOT (post-logMAR)	1.67±0.74
BCVA after HBOT ≤1.0 logMAR	30 (26%)
BCVA change (dif-logMAR)	-0.45±0.74
Clinically significant improvement (dif-logMAR≥0.3)	52 (46%)
Subjective improvement	66 (58%)

VA outcome

The results showed a significant difference between BCVA before HBOT (pre-logMAR) (2.12±0.74) and after HBOT (post-logMAR) (1.67±0.74) for p=0.000. Overall, 54 patients experienced a BCVA gain with HBOT, 56 patients did not experience any BCVA change and 4 patients worsened despite therapy. A clinically significant visual improvement after HBOT, dif-logMAR≥0.3, occurred in 46% (n=52) of the patients. About 58% (n=66) of the patients reported subjective VA improvement after the treatment. The percentage of those with final BCVA better than 1.0 logMAR was 26% (n=30). 

The results from adjusted regressions models (Table 2) showed that the number of HBOT sessions contributes to the explanation of the values of the three variables: post-logMAR, dif-logMAR, and clinically significant visual improvement (dif-logMAR≥0.3), together with age on post-logMAR, with supplementary treatments on dif-logMAR and with obesity on clinically significant visual improvement. The number of HBOT sessions was shown to be the most important variable in the explanation of the VA outcome. The more HBOT sessions patients received, the better their VA outcome. This includes higher clinically significant visual improvement, greater dif-logMAR scores, and lower post-logMAR values, as shown in Table 2. From the model adjusted on dif-logMAR, the number of HBOT sessions that allow to reach the minimum dif-logMAR≥0.3, was calculated (seven sessions). This means that in the sample a minimum of seven sessions were necessary to promote a clinically significant visual improvement. Age influenced post-logMAR values, with older patients exhibiting higher post-logMAR values. The increase in post-logMAR from the minimum age (22 years old) to the maximum age (92 years old) is 1.14 (0.016 by each year old). Supplementary treatments have been shown to reduce dif-logMAR values by 0.248 logMAR. The isolation of the effects of each supplementary treatment was not possible in this work, due mainly to the insufficient number of patients for each treatment. The logistic regression adjusted on clinically significant visual improvement showed significant effects of a number of sessions (positive) and obesity (negative). The odds ratio of HBOT sessions was 1.498 (95% CI 1.238-1.814), and the odds ratio of obesity was 0.335 (95% CI 0.125-0.899).

**Table 2:** Adjusted multiple regression models on post-logMAR, dif-logMAR (OLS), and clinically significant visual improvement (dif-logMAR≥0.3) (logistic) and respective coefficients (C), estimates (E), and probabilities (p) C: coefficient (post-logMAR and dif-logMAR); E: estimate (dif-logMAR≥0.3); n: number of cases; R2: squared multiple R; Sy: standard error of y *: Cox and Snell R-square, for multiple logistic regression; odds ratio of HBOT sessions is 1.498 and of obesity is 0.335 on clinically significant improvement (dif-logMAR≥0.3)

Regression models														
VA outcome (y)	Constant		Age		HBOT sessions		Supplementary treatments		Obesity		Model statistics			
	C/E	p	C	p	C/E	p	C	p	E	p	n	p	R^2^	S_y_
post-logMAR	1.082	0.043	0.016	0.015	-0.068	0.011	-	-	-	-	114	0.000	0.135	0.864
dif-logMAR	0.218	0.185	-	-	-0.106	0.000	0.248	0.045	-	-	114	0.000	0.240	0.650
dif-logMAR≥0.3	-2.805	0.000	-	-	0.404	0.000	-	-	-1.094	0.030	114	0.000	0.240^*^	-

The MANOVA analyses added only a significant effect of the variable CRS on post-logMAR values (p=0.026). Mean values were 1.84±0.89 for patients with CRS and 1.54±0.89 without CRS, which means an increase of 0.30 in the post-logMAR for patients with CRS at presentation compared with patients without CRS.

Last, both variables pre-logMAR and post-logMAR were significantly correlated (r=0.597), which means that changes in pre-logMAR to post-logMAR values, after HBOT, tend to keep the relative initial rank of data.

## Discussion

The results showed a significant positive impact of HBOT on VA in patients with CRAO, aligning with the findings from most previous studies [14-18]. Clinically significant VA improvement was observed in almost half of the patients (46%) following HBOT. This is consistent with the findings of a limited number of previous studies on CRAO patients treated with HBOT that applied similar inclusion and exclusion criteria. Coelho et al. [22] reported a 71% rate of clinically significant VA improvement in 14 patients and Hadanny et al. [14] reported 67% in 128 patients. The results of this study showed that HBOT revealed potential benefit even in patients with very poor VA at presentation (≥2.70 pre-logMAR) where half of the patients showed clinically significant VA improvement. VA at presentation, as an exclusion factor for HBOT, per se, should therefore be carefully weighed. Nevertheless, the degree of improvement experienced by the patients was limited. Mean BCVA after HBOT was 1.67±0.74 logMAR and only 26% (n=30) ended the treatment with a BVCA ≤1.0 logMAR. The published results in similar populations showed identical tendencies, BCVA after HBOT ranging from 1.39±0.94 logMar [22] to 2.15±1.07 logMAR [16].

It was found that VA outcome was related to the total number of HBOT sessions, patient’s age, obesity, supplementary treatments, and the presence of CRS at presentation. Among these factors, the total number of HBOT sessions had the most significant impact on VA outcomes. The higher the total number of sessions the better VA outcome. However, it is important to note that, according to the protocol, only patients who showed improvement would undergo additional sessions, extending beyond the initial five to six sessions. The median number of sessions completed in the sample was seven (HBOT equivalent length of 10.5 hours). Furthermore, it was observed that seven sessions were the minimum number associated with clinically significant visual improvement. It was reported in a meta-analysis of seven randomized controlled trials that the median number of HBOT sessions was three and according to the study’s findings, the most effective treatment length was over nine hours [18]. The unit’s current protocol contemplates performing a minimum of five to six sessions (HBOT equivalent length of 7.5 to 9 hours) for CRAO treatment. There are no guidelines recommending a CRAO HBOT protocol with an exact number of sessions. According to the Undersea and Hyperbaric Medical Society, the appropriate number of HBOT sessions depends on the severity and duration of a patient’s symptoms, as well as the degree to which those symptoms are responding to the HBOT treatments administered [12].

The mean patient’s age in the sample was expected from what is described in the literature [23], where the average age is in the 60s. Comorbidities and chronic medications, including obesity showed a prevalence similar to the Portuguese population in the same age group [24]. CRAO indicates end‑organ ischemia, often due to underlying atherosclerosis. Older age and obesity are well-known atherosclerotic risk factors. But beyond being risk factors to CRAO, the results showed less improvement of VA with aging and when obesity was present. So, there is the suggestion that age and obesity are potential VA predictors in CRAO. It must be taken into consideration that more than half of patients after CRAO had at least one undiagnosed vascular risk factor [25] but simultaneously, obesity and older age are objective diagnoses and probably its data is the closest to the true prevalence.

Recently CRS was proposed as a biological marker representing complete anoxia and irreversible infarction of the macular area [14]. Hadanny et al. [14] reported a linear correlation between the presence of a CRS and the final BCVA. In their series, the presence of a CRS decreased the gain in logMAR by 0.787. In this study despite the presence of a CRS did not have a significant effect on the adjusted regression models, it was found to have a significant effect on the BCVA after HBOT, with mean logMAR values of 1.54±0.89 and 1.84±0.89, without and with CRS, respectively.

Supplementary treatments showed a negative influence on VA outcome (dif-logMAR). The available evidence suggests that supplementary treatments for CRAO often fail to improve visual outcomes [1,4], except for thrombolysis which may be considered in certain situations as a viable treatment option [5]. However, in the sample, only five patients (4%) received fibrinolytic therapy. There are some concerns regarding the potential risks and complications associated with certain conservative treatments [1,5]. However, there is limited specific evidence to indicate that these treatments worsen visual outcomes [26]. Due to the wide variety of these treatments, their different combinations and the low number of patients per treatment it was not possible to isolate the effect of each treatment in this study. In the presence of this heterogeneity, the conclusions on this topic are therefore unclear. However, this underscores the importance of cautious application of supplementary treatments and the need for further research.

The time delay from symptoms to treatment has been described as a VA outcome predictor in CRAO [6,14]. However, there was no correlation found between time delay from symptoms to treatment and VA outcome. To reduce the risk of bias, a second analysis was performed excluding the patients who woke up with symptoms, and whose timing from starting symptoms was not precise, but no significant difference was observed. Other factors that can influence VA in CRAO include anatomical variations, type, degree of occlusion and residual perfusion, patient factors, and the etiology of the occlusion [1]. As the time of progression from ischemic to infarcted retina depends on various factors and cannot be fully predicted in humans, time itself should not be considered a predictor of treatment efficacy; nonetheless, it remains an important factor. For instance, a study highlighted that patients treated within the first 4.5 hours had a higher rate of partial or full visual recovery compared to those treated later [6]. Some other studies, including a review of treatment options for CRAO, indicated that to be effective, treatment should be given within six hours of ischemia onset [26]. Others found that HBOT might still be beneficial up to 12 hours post-occlusion [27]. In summary, the consensus in the literature is that the earlier the treatment for CRAO, the better the chances for visual recovery [5,6] with the most critical period being within the first 4.5 to 6 hours. It was found a mean time delay from symptoms to treatment of 12 hours, which may explain the limited VA gain experienced by the patients. Other studies show improved VA from HBOT-initiated treatment within 6 to 12 hours of visual loss [14,28,29]. However, 12 hours is a substantial mean time delay, considering that CRAO is treated as an emergence in the Hyperbaric Unit. As the vast majority (81%) of the CRAO patients were referred from other hospitals it was investigated its possible impact in the time delay to treatment with HBOT. The results showed that the hospital of referral (Hospital Pedro Hispano versus other hospitals) or the distance between the hospital of referral and Hospital Pedro Hispano (≤15km versus >15 km) didn’t show a relation with time delay from symptoms to treatment. These results suggest that the longest delay occurs at the patient’s presentation to urgent care or/and in-hospital until arrival to the unit.

The reported subjective improvement was slightly higher than the objective clinically significant VA improvement (56% versus 48%), presenting, both variables, with a significant association coefficient (“Phi”=0.710). The majority of CRAO patients have poor VA, and most VA tests do not cover accurate measurements in the lower ranges of vision [21]. Then the subjective evaluation of the improvement can suggest a difference that is noticed by the patient but cannot be measured. Subjective evaluations could, therefore, complement the assessment of visual improvement in CRAO patients and serve as an indicator of patient satisfaction with HBOT.

HBOT, as a non-invasive treatment, showed significant effects on VA outcomes for CRAO patients, beyond its low adverse effects, in 6% (n=7) of the CRAO patients, which were mild and reversible. However, this study, like other similar, has limitations, most of which are inherent to its retrospective design, for instance, the control of how the data were originally collected; the data recording practices across different time periods, practitioners, or institutions; the absence of control group due to ethical reasons, since CRAO is an approved indication for HBOT. Further research is necessary to establish well-defined prognostic markers for assessing the benefits and risks for individual patients, as well as to emphasize the safety and effectiveness of HBOT. Patient delay to the Hyperbaric Unit is a significant concern in the sampled population, highlighting issues not only with public awareness of this entity but also with in-hospital response. Developing strategies to enhance public awareness of CRAO, such as through public health campaigns, and to improve HBOT knowledge among healthcare professionals, like creating an emergency protocol for managing in-hospital CRAO cases, is essential.

## Conclusions

HBOT was shown to be safe and beneficial for VA outcomes in patients with non-arteritic CRAO. The number of HBOT sessions has proven to be the most important variable for VA enhancement, with a minimum of seven sessions required for clinically significant visual improvement. Patient factors such as age, obesity, and the presence of CRS at presentation also influence VA outcomes. Strategies to promote and expand the use of HBOT in CRAO patients should be implemented. This may ultimately provide more robust evidence regarding its efficacy, safety, and potential prognostic indicators.
